# White Light Spectroscopy Characteristics and Expansion Dynamic Behavior of Primary T-Cells: A Possibility of Online, Real-Time, and Sampling-Less CAR T-Cell Production Monitoring

**DOI:** 10.3390/bios15040251

**Published:** 2025-04-15

**Authors:** Bruno Wacogne, Maxime Brito, Clémentine Gamonet, Alain Rouleau, Annie Frelet-Barrand

**Affiliations:** 1 CNRS, Institut FEMTO-ST, Université Marie et Louis Pasteur, 25000 Besançon, France; alain.rouleau@femto-st.fr (A.R.); annie.frelet-barrand@femto-st.fr (A.F.-B.); 2 Centre Hospitalier Universitaire de Besançon, Centre d’Investigation Clinique, INSERM CIC 1431, 25030 Besançon, France; 3 Etablissement Français du Sang Bourgogne-Franche Comté (EFS-BFC), 8 Rue Jean-François Xavier Girod, 25000 Besançon, France; maxime.brito@efs.sante.fr (M.B.); clementine.gamonet@efs.sante.fr (C.G.)

**Keywords:** CAR T-cell, white light spectroscopy, cell concentration monitoring, advanced therapy medicinal product, quality control

## Abstract

The production of advanced therapy medicinal products (ATMP) is a long and highly technical process, resulting in a high cost per dose, which reduces the number of eligible patients. There is a critical need for a closed and sample-free monitoring system to perform the numerous quality controls required. Current monitoring methods are not optimal, mainly because they require the system to be opened up for sampling and result in material losses. White light spectroscopy has emerged as a technique for sample-free control compatible with closed systems. We have recently proposed its use to monitor cultures of CEM-C1 cell lines. In this paper, we apply this method to T-cells isolated from healthy donor blood samples. The main differences between cell lines and human primary T-cells lie in the slightly different shape of their absorption spectra and in the dynamics of cell expansion. T-cells do not multiply exponentially, resulting in a non-constant generation time. Cell expansion is described by a power-law model, which allows for the definition of instantaneous generation times. A correlation between the linear asymptotic behavior of these generation times and the initial cell concentration leads to the hypothesis that this could be an early predictive marker of the final culture concentration. To the best of our knowledge, this is the first time that such concepts have been proposed.

## 1. Introduction

### 1.1. Context

Advanced Therapy Medicinal Products (ATMPs) are innovative medicines derived from genes, cells or tissues and intended for the treatment of chronic, degenerative or life-threatening diseases [1]. These therapies rely on genetic modification or tissue engineering to acquire novel physiological or biological properties, including regenerative properties. The production of ATMPs requires complex and costly technologies such as cell sorting, amplification, genetic transduction and activation [2,3,4]. The entire process is carried out under strict environmental conditions with multiple quality checks over a period of up to 10 days. As a result, high production costs limit widespread access to these promising treatments. Current equipment often lacks integrated online monitoring technologies, tracking only basic parameters such as temperature, pH or dissolved oxygen via sterile probes placed in the bioreactor. Recognizing this gap, the FDA launched the Process Analytical Technology (PAT) initiative in 2004 to foster innovation in real-time monitoring for biopharmaceutical manufacturing.

### 1.2. Commercial Methods

Historically, cell concentration has been determined using direct microscopic counts, such as Malassez cells. While widely used, this method has the drawback of manual counting, low reproducibility, and limited representativeness due to the small sample volumes analyzed. Automated alternatives are now modifying cell counting. Systems such as the LUNA^TM^ (LOGOS BIOSYSTEMS; [5,6,7]) require 10 µL of suspension and use conventional imaging and processing. Similarly, IPRASENSE offers devices such as NORMA and CYTONOTE that use lensless imaging to improve accuracy [8]. NORMA requires only 10 µL of sample, while CYTONOTE is optimized for adherenT-cells in larger volumes. Advanced systems such as INCUCYTE^®^ (SARTORIUS [9,10]) and HoloMonitor ^®^ (PHI [11]) combine in situ microscopy with holographic imaging, enabling cell counting directly within the incubation environment and across multiple volumes, including 96-well plates for high-throughput applications. Despite their easy use, these technologies are challenging to integrate into real-time, and closed systems.

### 1.3. Alternative Techniques

Other methods exist for the qualification of cells and subcellular components. Some rely on ligand–analyte interactions for biological capture, such as enzyme-linked immunosorbent assay (ELISA) [12,13], surface plasmon resonance [14,15], or quartz crystal microbalance techniques [16,17]. However, these approaches rely on biochemical interfaces and require frequent surface regeneration, making them difficult to adapt to real-time systems. Non-biochemical approaches, such as impedance spectroscopy (or dielectric spectroscopy), are widely used to monitor mammalian cell cultures [18]. By applying an alternating electric field, the cells are polarized, allowing for quantification with advantages such as in situ analysis and rapid measurements. However, this method requires calibration and its accuracy decreases during the stationary growth phase [19].

Spectroscopic techniques [20], including Raman spectroscopy, have been used for cell characterization and identification, either directly in situ [21] or with surface-enhanced Raman scattering [22,23,24]. These techniques have applications ranging from cell culture quality control [23] to pathogen detection [25]. Although highly accurate and enabling cell identification, such data may exceed the requirements for routine cell monitoring, especially in the context of CAR T-cell production. Flow cytometry is another versatile option, allowing for simultaneous cell counting and assessment of biological properties [26,27,28]. However, they often require additional sample preparation, such as fluorescence labeling [29].

### 1.4. T-Cell Lines Versus Primary T-Cells

On the one hand, the purpose of a primary T-cell is to combat a pathogen. When it is activated (by CD3 and CD28 commitment), this recognition of a foreign pathogen is simulated. The primary T-cells multiply in order to destroy the pathogen. But this activation is transient and must be stopped to prevent the immune system from going into overdrive. Primary T-cells are therefore ‘programmed’ to multiply for a time, respond strongly to stimulation, and then gradually stop proliferating. After proliferating a lot, they can become ‘exhausted’. During their lifetime, T-cells can be stimulated several times (and therefore undergo several cycles of proliferation), but, after a while, they become senescent/apoptotic and no longer respond to stimulation. The growth rate slows down, resulting in an increasing generation time.

On the other hand, T-cell lines are immortalized cells. They do not function at all like primary T-cells, since, by definition, they no longer have any control mechanisms. T-cell lines, as other cell lines, are derived from primary cells but have been subcultivated and/or immortalized to achieve “cancer-like” features. As a result, they can proliferate continuously without specific stimulation and without senescence. In this case, the growth rate remains constant, as well as their generation time.

More information can be found in [30,31], although the dynamical aspects are not a priority.

### 1.5. Current Needs and Proposal

Most currenT-cell counting methods rely on sampling small and potentially unrepresentative volumes. Furthermore, this sampling can be the source of contamination. Indeed, there is a need for methods that can be directly applied to a sample-free, closed-loop system and that can also provide real-time cell concentration evolution. We have recently proposed a white light spectroscopy technique that could meet these requirements. We demonstrated that CEM-C1 cell lines could be efficiently monitored by analyzing the shape of their absorption spectra and its evolution with concentration. CEM-C1 showed exponential expansion behavior over the reported 1-week experiments, indicating that the generation time was constant over time [32].

In this paper, we apply the concept to T-cells from healthy donors and show that both their absorption spectra and expansion dynamics differ from those of CEM-C1 cell lines. The next section presents the “Materials and Methods” of the present study. Section 3 presents the experimental results. The T-cell concentration is measured spectrally by fitting the shape of the absorption spectra with a mathematical function, the elaboration of which is presented in Section 3.1. This function is used to measure T-cell concentration during cultures performed with human T-cells from blood donations (Section 3.2). A method for describing the non-exponential growth of cultured T-cells is proposed in Section 3.3. The concept of instantaneous generation time is proposed in Section 3.4 to address the non-exponential behavior of T-cell culture. The results are discussed in Section 4. The present paper proposes proofs of concept in a laboratory environment. Considerations for potential integration of the method into CAR T-cell production units will also be discussed. Finally, a conclusion is proposed.

## 2. Materials and Methods

**Figure 1:**
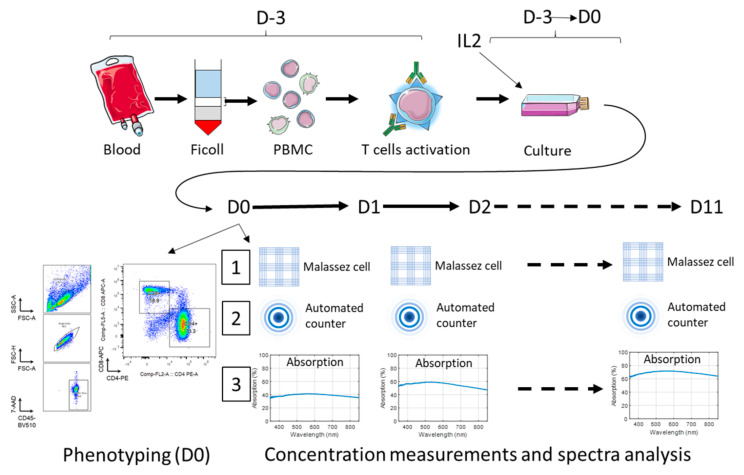
General experiment protocol.

The general experimental protocol is presented in Figure 1. The reagents and equipment are summarized in Table 1.

### 2.1. Peripheral Blood Mononuclear Cell (PBMC) Extraction, Platelet Removal and T-Cell Activation

This protocol was performed at D-3 (D0 being the beginning of concentration measurements).

PBMCs were isolated from a blood sample collected from the tubing of a platelet donation kit by cytapheresis using a density gradient (Ficoll).

Furthermore, 12 × 10^6^ PBMC were activated by TransActTM at 5 µL per 10^6^ PBMC and directly seeded at 2 × 10^6^ PBMC.mL^−1^ in RPMI-HS (8%)-PS (1%)-IL-2 (0.4 µL.mL^−1^). PBMCs were incubated for 3 days at 37 °C, 5% CO until “Day 0” to amplify the T-cells.

### 2.2. T-Cell Seeding and Phenotyping at D0

At D0, cell counts and viability assessments were performed by the Trypan blue/Malassez cell method. In addition, 5.4 × 10^6^ T-cells were collected and resuspended in (RPMI-HS (8%)-PS (1%)-IL-2 (0.4 µL.mL^−1^) at 0.15 × 10^6^ Tcells.mL^−1^, and 3 mL of this cell suspension was transferred into a spectroscopy cuvette for absorption spectrum measurement. The remaining T-cells were transferred to a T75 culture flask and incubated at 37 °C, 5% CO_2_.

### 2.3. Absorption Spectra Acquisitions

Absorption spectra were acquired using the experimental setup presented in [32,33]. It mainly consisted of a white light source, a spectrometry cuvette holder and a compact UV/VIS spectrometer. Transmission spectra were acquired using the OceanView software, transformed into absorption spectra and mathematically processed using the MatlabTM software (version R2020b). Data fitting was performed using the Curve Fitting toolbox of the MatlabTM R2020b software. The spectra were acquired with 3647 data points between the 177 nm and 892 nm wavelengths. They were truncated to retain the range between the 350 nm and 850 nm wavelengths where the signal-to-noise ratio is higher.

### 2.4. T-Cell Culture Experiments

T-cells were cultured for 11 days and the concentrations were measured in 3 ways: with a Malassez cell using trypan blue (50%), with an automated cell counter, and by processing absorption spectra (see below). The experiments were performed 1 time with 6 different blood donations. The concentrations were measured on the days indicated in Table 2; a total of 9 measurements were performed except for donation #6. For donations #4 and #5, the concentrations were measured using Malassez cells only on D4 due to experimental issues. A total of 53 triple concentration measurements were performed on the 6 donations.

The cells were maintained at concentrations corresponding to the optimal culture concentration ranges. If the concentrations were too high, the cell volumes were periodically collected and resuspended in the culture medium to maintain a concentration between 0.2 × 10^6^ Tcell.mL^−1^ and 1 × 10^6^ Tcell.mL^−1^. Every 2 or 3 days, the cells were resuspended at 0.2 × 10^6^ TcellmL^−1^ in RPMI-HS (8%)-PS (1%). Table in Section 3.2 summarizes the days on and dilutions at which the cells were resuspended.

### 2.5. Dilution Ranges

Cell concentration measurements based on white light spectroscopy were performed by analyzing the shape of the absorption spectra [32]. Assuming that donor T-cells may interact with light differently that CEM-C1 T-cell lines, dilution ranges were made to obtain spectra measured at concentrations ranging from 7 × 10^4^ to 1.2 × 10^6^ Tcells.mL^−1^. Dilution ranges were made at D4 and D7 (donations #1 and #2), D4, D7 and D11 (donation #3), D7 and D11 (donation #6), and D9 for a donation not included here due to missing spectrometry measurements at D0 and D1. A total of 73 spectra and corresponding concentrations were used to establish dilution ranges.

## 3. Results

In this section, CEM-C1 refers to T-cell lines and T-cell refers to cells obtained from healthy donors.

### 3.1. Description of the Primary T-Cell Absorption Spectra Shapes

Previously, the absorption spectra of CEM-C1 cell lines were described by two Gaussian functions whose parameters depended on the cell concentration [32]. Concentrations were measured with an accuracy of about ±4.5%, sufficient for the intended applications and better than that obtained with an automated counter due to the representativeness of the samples collected for automated counting [33]. The function describing the shapes of the CEM-C1 spectra as a function of concentration was used to calculate the T-cell concentrations of the dilution ranges described in Section 2.5. Concentrations calculated with this function were systematically lower than the actual T-cell concentrations. A function corresponding to the optical properties of T-cell suspensions was then calculated using the method described in [32].

#### 3.1.1. Determining the Primary T-Cell Absorption Spectra Shapes

**Figure 2:**
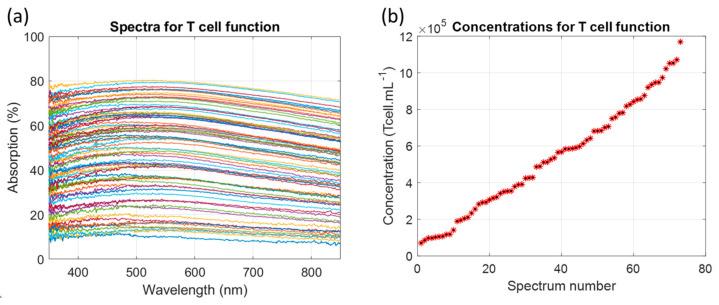
Absorption spectra and corresponding concentrations of dilution ranges of T-cells. (**a**) Absorption spectra (each color represents one concentration). (**b**) Corresponding concentrations measured with a Malassez cell. n = 73.

Spectra of the dilution ranges were recorded and the corresponding concentrations were measured using a Malassez cell (Figure 2).

The T-cell concentrations ranged from 7 × 10^4^ to 1.17 × 10^6^ Tcell.mL^−1^. Absorption spectra maxima ranged from 11% to 80%. Comparisons between CEM-C1 and primary T-cell absorption are discussed in Section 4.1 and Section 4.2. The method for determining the function describing the shape of the cell absorption spectra as a function of concentration was published earlier [32]. It was applied to dilution ranges of primary T-cells. In summary, the general shape of T-cell absorption spectra was represented in its general form by two Gaussian functions (Equation (1)).

(1) $${Abs}_{Tcell}\left(\lambda ,C\right)=a_{1}\left(C\right)exp\left\{-{\left(\frac{\lambda -b_{1}(C)}{c_{1}(C)}\right)}^{2}\right\}+a_{2}(C)exp\left\{-{\left(\frac{\lambda -b_{2}(C)}{c_{2}(C)}\right)}^{2}\right\}$$

In Equation (1), $a_{i}\left(C\right)$, $b_{i}(C)$ and $c_{i}\left(C\right)$ represent the “a priori” concentration-dependent amplitude, center and width of Gaussian “i”. The absorption function was established in two main steps. A series of successive fits with two Gaussian functions allowed for the determination of constant Gaussian parameters and concentration-dependent parameters. Then a minimization algorithm was used to calculate the final parameters of the spectra shape function, as presented before [32]. It was found that only the amplitude and the width of the first Gaussian depended on concentration, as was also the case for CEM-C1 T-cell lines. Furthermore, the $a_{1}\left(C\right)$ and $c_{1}\left(C\right)$ sub-functions exhibited the same mathematical forms. Indeed, the equations describing the shape of CEM-C1 T-cell lines and primary T-cells have the same form; only numerical parameters differ between them. Finally, the primary T-cell absorption spectra are described by Equation (2) with parameters obtained after the step-by-step and the minimization stages described in [32] (Table 3).

(2) $${Abs}_{Tcell}\left(\lambda ,C\right)=100\left(1-10^{-p1a1.C}\right)exp\left\{-{\left(\frac{\lambda -b_{1}}{p1c1.C^{p2c2}}\right)}^{2}\right\}+a_{2}exp\left\{-{\left(\frac{\lambda -b_{2}}{c_{2}}\right)}^{2}\right\}$$

The next step was to test the T-cell absorbance function on the dilution range spectra.

#### 3.1.2. Measuring Dilution Ranges with the T-Cell Absorption Function

**Figure 3:**
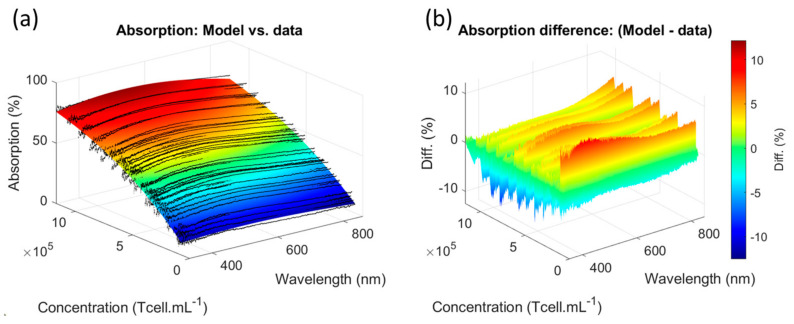
Comparison between T-cell absorption function and experimental dilution ranges spectra. (**a**) Function vs. experimental data. (**b**) Difference between absorption function and spectra.

The primary T-cell absorption function was compared to the experimental dilution range spectra (Figure 3).

The experimental spectra were equally distributed on both sides of the absorption function (Figure 3a). The difference between the experimental absorption and the spectrum function did not exceed ±5% (Figure 3b). It was observed that this difference tended to increase with decreasing concentrations. The accuracy of the spectral measurement compared to automated counting is discussed in Section 4.3.

**Figure 4:**
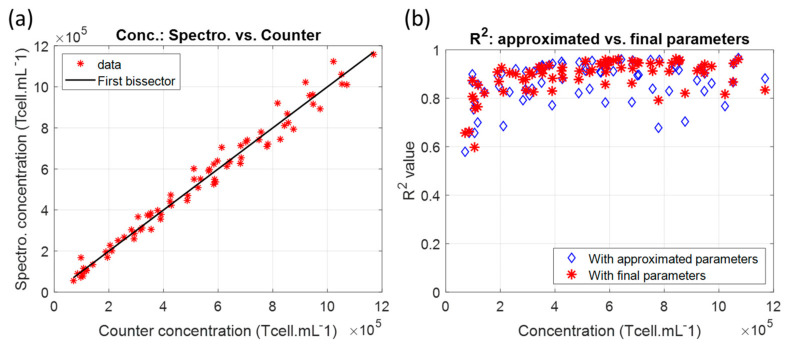
Measuring concentration in dilution ranges using the absorption function. (**a**) Spectral vs. automated counting. (**b**) R^2^ values of spectra fittings with absorption function.

The absorption function was used to fit the spectra of the dilutions (Figure 4a). The difference between spectrally measured and automatically counted concentrations did not exceed ±5%, as suggested by Figure 3b. This accuracy has been observed previously with CEM-C1 cell lines [32]. The goodness of fit was calculated (Figure 4b). R^2^ values were always above 0.8, except at a low concentration, where R^2^ decreased to 0.6. Nevertheless, the measurement accuracy remained in the accepted ±5% range even for concentrations for which the R^2^ around 0.6 is very low. This is discussed in Section 4.3. It should be noted that the R^2^ values obtained with the approximated parameters were more scattered than those obtained with the parameters calculated after minimization.

The T-cell concentration function was then used in experiments conducted with T-cells issued from six different donations.

### 3.2. Primary T-Cell Culture Monitoring

**Figure 5:**
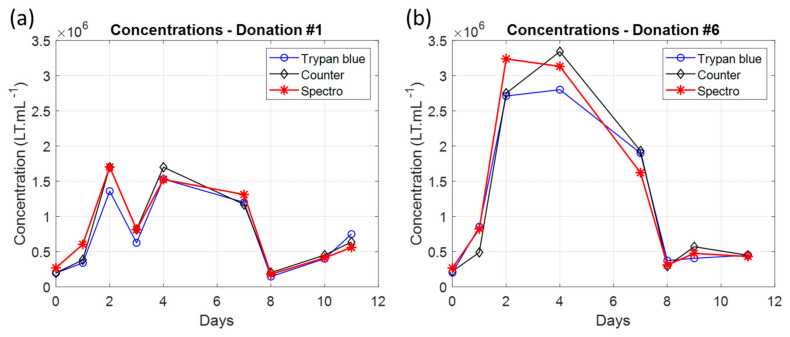
Examples of T-cell culture with concentration measurements performed with a Malassez cell, an automated counter and a spectrometer. (**a**) Donation #1. (**b**) Donation #6.

T-cells issued from six blood donations were cultured over 11 days according to the protocol described in Section 2.4. The T-cell concentration was measured on a daily basis using a Malassez cell, an automated counter, and the use of absorption spectra. Examples of experiments for representative experiments are shown in Figure 5.

The concentrations measured spectrally were consistent with the concentrations measured by conventional means. Spectral measurements were found to be accurate between 7 × 10^4^ and 1.2 × 10^6^ Tcell.mL^−1^ (Section 3.1.2). For higher T-cell concentrations, the cells transferred to the spectroscopy cuvettes were diluted to obtain measurements within the accurate spectral range. The suspensions were then diluted by two on D2 for donations #4 and #5. They were diluted by two, four and two on D2, D3 and D7, respectively, for donation #6.

As explained in Section 2.4, T-cells were maintained at concentrations corresponding to the optimal culture concentration ranges, i.e., between 0.2 × 10^6^ and 3 × 10^6^ Tcell.mL^−1^. The T-cells were regularly collected and resuspended in volumes of culture medium to reduce their concentration. Table 4 summarizes the dilution factors and the days of dilutions for each experiment. They were calculated from concentration measurements made with a Malassez cell.

The expansion fold is a measure that describes how much the number of T-cells increases between its initial value and what it should be if no resuspension was performed and if the volume of culture medium was gradually adjusted to allow the cells to grow at a suitable concentration. The fold was calculated for each concentration at the mean of the measurements as follows. At D0, the fold is set to 1 for all assays. For each method, the fold at measurement day “i” was calculated as follows.

(3) $${Fold}_{Method}\left(i\right)=\frac{C_{Method}(i)}{C_{Method}(i-1)}\times F_{dilution}\left(i\right)\times {Fold}_{Method}(i-1)$$

**Figure 6:**
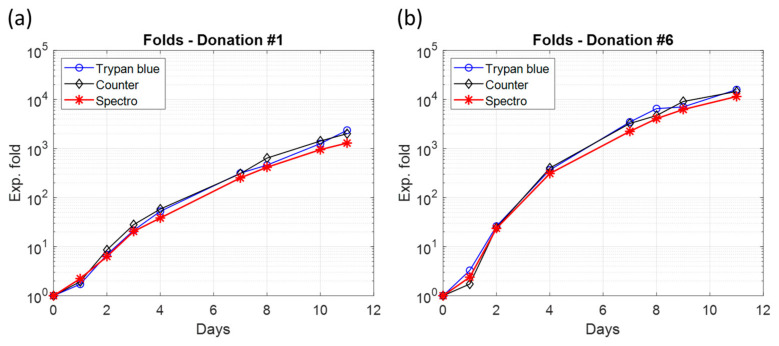
Examples of expansion folds obtained for each concentration measurement method. (**a**) Donation #1. (**b**) Donation #6.

In Equation (3), $C_{Method}(i)$ is the concentration measured on day “i” with the measurement method *Method* and $F_{dilution}\left(i\right)$ is the dilution factor specified in Table 4. Folds were calculated for all donations and examples of folds calculation for donations #1 and #6 are shown in Figure 6.

The final expansion fold values of all measurement methods were calculated and averaged (Table 5). The final folds for experiments #4 and #5 were much lower, probably due to the use of an almost expired activation antibody. Conversely, the final fold of experiment #6 was extremely high, as is sometimes the case. In all cases, the variations in the final fold values between experiments highlighted the fact that primary T-cells grow quite differently from one donor to another one, which was, of course, not observed with CEM-C1 cell lines.

The folds showed non-linear behavior on the logarithmic plots (Figure 6, as would be expected for exponential cell populations, i.e., with a constant generation time (time required for a cell population to double). This aspect was investigated.

### 3.3. Non-Exponential T-Cell Multiplication

The folds were not linear when drawn with a logarithmic vertical scale. They all showed a convex part between D0 and D3 and a concave part between D3 and D11. This meant that the concentrations did not develop exponentially when plotted with a linear vertical scale. In other words, the time required for a cell population to double decreased over time. Therefore, an exponential function could not be used to describe the increase in cell concentration because it would imply a constant generation time, i.e., a linear progression in a logarithmic vertical scale.

**Figure 7:**
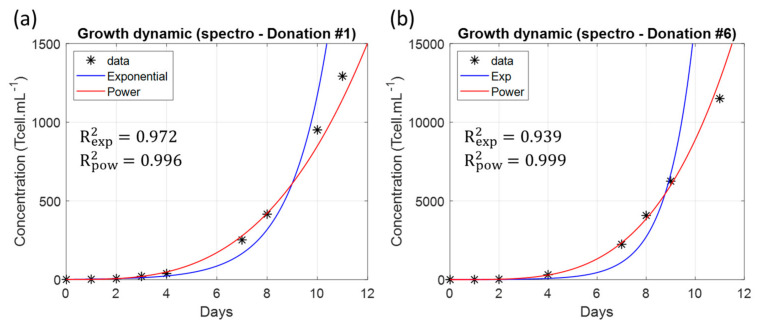
Examples of expansion folds fitted with either an exponential or a power function with corresponding R^2^ values. (**a**) Donation #1. (b) Donation #6. (**a**) and (**b**) are not drawn to the same scale. Black stars: concentrations spectrally measured, blue curves: exponential fitting and red curves: power fitting.

It was found that a power function could be efficiently used to describe the fold evolution (Figure 7).

On spectrometry data, the fitting R^2^ using a power function was always greater than 0.99, while it was always less than 0.97 when using an exponential function. The power function used to describe the fold evolution is given below.

(4) $$fold\left(t\right)=fold\left(0\right)+at^{b}=1+at^{b}$$

Parameters ‘a’ and ‘b’ were calculated for all experiments and are reported in Table 6 for spectrometric data only. The R^2^ values of the power-law fits are also reported. However, precautions were taken in fitting the data, as discussed in Section 4.4.

**Figure 8:**
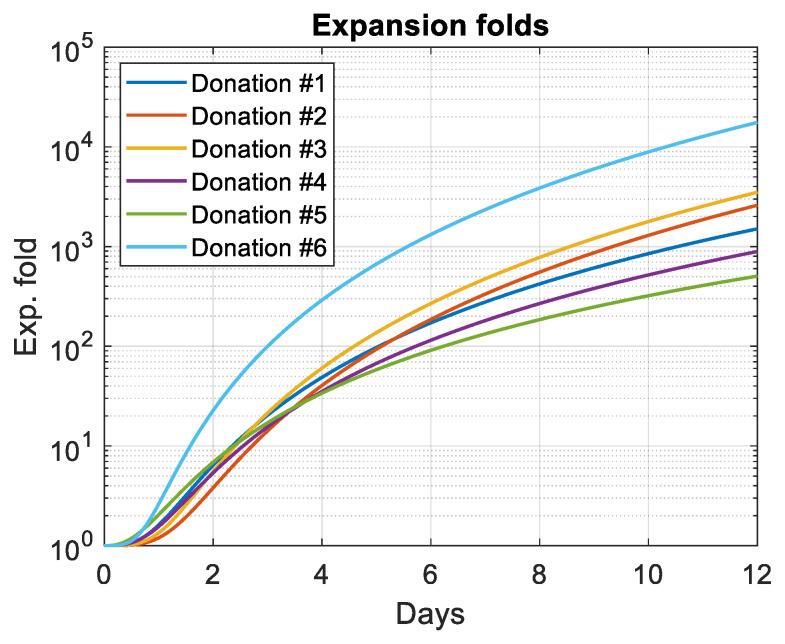
Expansion folds calculated using Equation (6) and parameters in Table 6.

Fitted folds were then reported for each experiment based on spectrometry data (Figure 8).

As mentioned above, the non-exponential evolution of the folds indicated that the multiplication rate of primary T-cells decreased over time. This means that the concept of generation time as conventionally defined does not apply to donor-derived T-cells. To the best of our knowledge, this is the first time that the non-exponential growth of T-cells is described mathematically.

### 3.4. Proposal of an Instantaneous Generation Time (IGT)

For any time *t*_1_, it is possible to determine the time *t*_2_ when the fold has doubled compared to its value at *t*_1_.

(5) $$fold\left(t_{2}\right)=1+a{t_{2}}^{b}=2\left(1+a{t_{1}}^{b}\right)$$

Time *t*_2_ can then be written as follows.

(6) $$t_{2}={\left(\frac{1+2a{t_{1}}^{b}}{a}\right)}^{1/b}$$

The quantity $\left\{t_{2}-t_{1}\right\}$ represents the generation time that T-cells would have had if they had continued to proliferate at a rate given by the tangent to the fold at time t_1_. This means that an Instantaneous Generation Time can be defined at any time “*t*” as follows.

(7) $$IGT\left(t\right)={\left(\frac{1+2at^{b}}{a}\right)}^{1/b}-t$$

**Figure 9:**
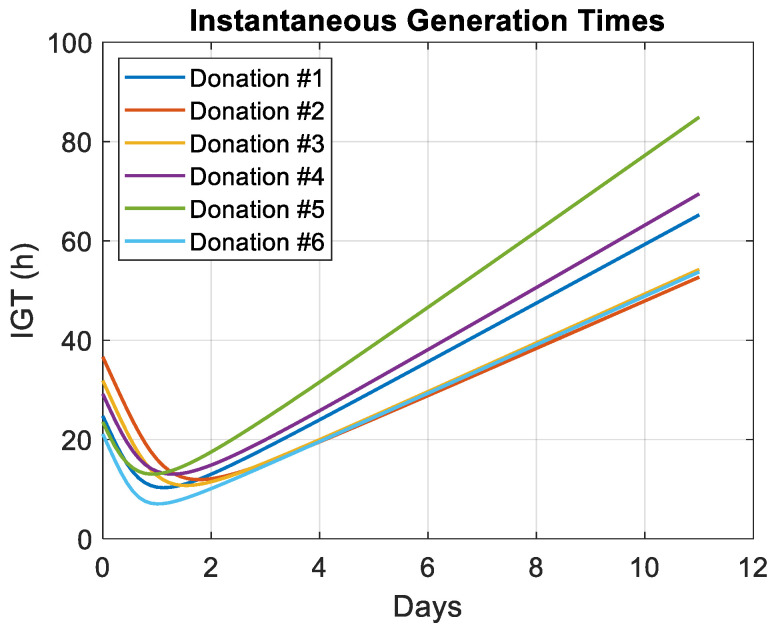
Instantaneous Generation Times (IGTs) calculated using Equation (7) for all experiments. Vertical scale in hours.

IGTs were calculated for all donations (Figure 9).

All IGTs decreased during the first day to reach their minimum values around D1. However, what seemed to be more informative were the slopes of the IGTs when growth had settled down (Table 7).

In fact, the slopes represent how many more hours are required each day for a T-cell population to double. It was found that, with the exception of experiments #4 and #5 where an activation antibody problem was suspected, human T-cells reduce their potential generation time by 5.4 to 6.5 h each day of culture. This is also, to the best of our knowledge, the first time that the concept of IGT is proposed.

## 4. Discussion

### 4.1. Primary T-Cell vs. CEM-C1 Optical Absorption

**Figure 10:**
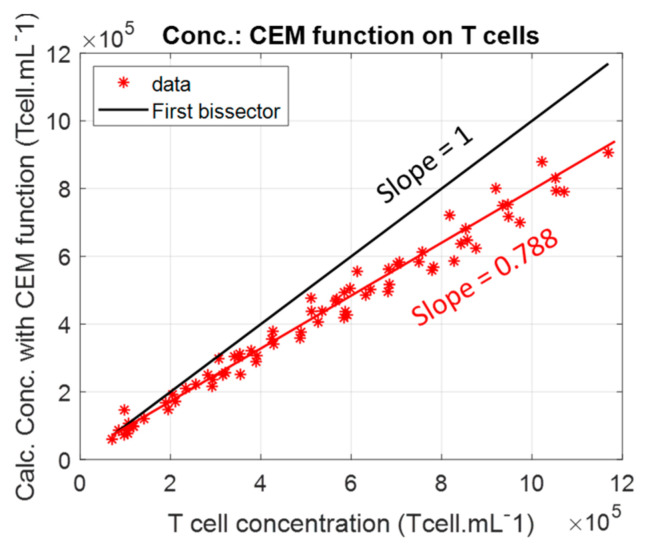
Calculating primary T-cell concentration using the CEM-C1 spectral shape function.

Figure 2a shows the absorption spectra of T-cell dilutions. The concentrations ranged from 7 × 10^4^ to 1.17 × 10^6^ Tcell.mL^−1^. The corresponding absorption maxima values ranged from 11% to 80%. The dilution ranges of CEM-C1 cell lines were previously used with concentrations ranging from 7 × 10^4^ to 1.15 × 10^6^ CEM.mL^−1^ corresponding to absorption maxima between 10% and 85% [32]. This suggests that primary T-cells absorb less light than CEM-C1. This is confirmed when the concentration is calculated from the spectra of Figure 2a using the shape equation of CEM-C1 (Figure 10).

This showed that T-cells absorbed 22.2% less light than CEM-C1 cell lines.

### 4.2. Concerning T-Cell Spectra Shape Function

Successive fits were used to determine constant and concentration-dependent Gaussian coefficients. The result was that only the amplitude and width of the first Gaussian evolved with concentration. This was previously observed in CEM-C1 [32]. Furthermore, the concentration dependence of these T-cell subfunctions could be fitted with the same function as for CEM-C1. As a result, the functions describing the shapes of the absorption spectra of CEM-C1 and T-cells had exactly the same mathematical form, but the constant parameters differed between the two species. This is not surprising since, in both cases, the function describes the spectral shape of cells of the same type. Only their origin (donor or cell line) is different, which explains why the functions describing their absorption spectra are similar but include different sets of parameters. This has already been observed while studying ESKAPEE bacteria for which a unique equation was used to describe their absorption spectra with sets of parameters specific to each bacterium [34].

**Figure 11:**
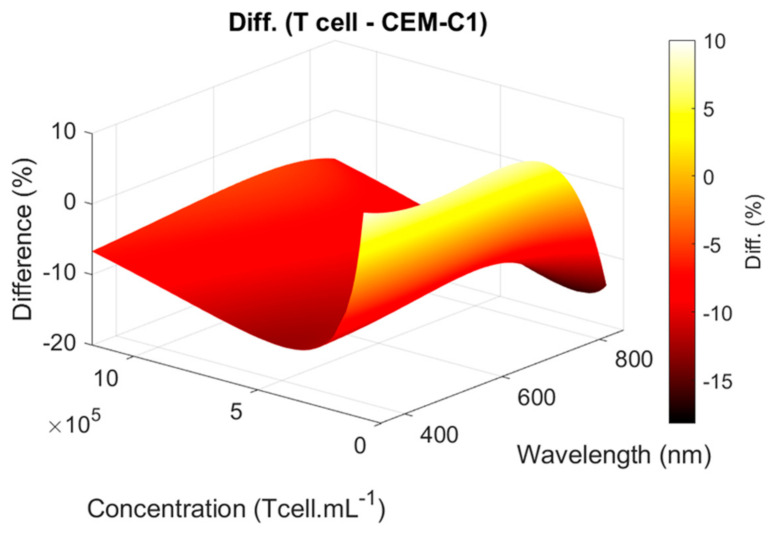
Difference between T-cell and CEM-C1 spectra functions.

The difference between primary T-cell and CEM-C1 functions was plotted (Figure 11). The CEM-C1 function was always 5% to 10% greater than the T-cell function, except at very low concentrations. This is consistent with Figure 10.

The parameters in Equation (2) do not form an orthogonal basis for the description of the spectra. Consequently, many sets of parameters that efficiently model the surface formed by the (concentration, wavelength, absorption) triplets can be defined. The coefficients in Table 3 were calculated by minimization with the initial values given the step-by-step process [32]. A second run of the minimization algorithm starting with the values in Table 3 would produce a different set of parameters, the latter being as efficient as the previous ones in describing the above surface. Since each parameter does not correspond to a biophysical property, any set that leads to an accurate estimation of the surface is acceptable. Concentrations calculated with any acceptable set of parameters would in any case be extremely close, sometimes even indistinguishable from each other.

More generally, studying the shape of the absorption spectra can be used to monitor the growth of any type of cell as long as a suitable function is determined. Furthermore, it can be used to monitor different species independently and simultaneously by using the law of OD additivity [32]. It was also proposed for ESKAPEE bacteria monitoring where a mixture of cells and bacteria was described [34]. Finally, the shape of the spectra can potentially be used for detecting contaminations of cell cultures [3,4].

### 4.3. Concerning Concentration Measurements’ Accuracy

Primary T-cell concentrations were measured with an accuracy of approximately ±5%. This is very similar to what has been observed with CEM-C1 cell lines, with an accuracy of ±4.5% [32]. The accuracy obtained when using a Malassez cell ranges from ±5% to ±10% and is human-dependent. Using a LUNA-II^TM^ automated cell counter (Logos Bio-systems^®^, Republic of Korea, supplier France), an accuracy of ±8% was observed [33]. These accuracies, which are lower than those reported by the manufacturer, are due to several factors, which are discussed below. It could be assumed that spectral measurements have the same accuracy as the conventional measurement method. In fact, the potential accuracy of spectral measurements is better. Basic plastic spectroscopy cells were used in these experiments. A complementary study (results available upon request) showed that the dispersion of the absorption measurements due to the plastic cuvette alone is ±2.5%. Knowing that uncertainties add up, it is concluded that spectroscopic measurements are more accurate than conventional methods. In fact, cell-culture-monitoring studies performed with a single spectroscopy cuvette throughout the experiment showed an accuracy of 3.3% [32].

The reason for this is twofold: the suspension volume sampled for concentration measurements and the number of cells counted by either measurement method. Consider a suspension with a concentration of 10^6^ cell.mL^−1^, 10 µL is usually sampled for Malassez cell measurements, but 1 µL is used for counting under the microscope. In the best case, when all cells are counted visually, a maximum of 10^3^ cells are counted. In most cases, only 10^2^ cells are actually counted. The automated counter used in this study uses 20 µL of the sample, but the examined volume is 1 µL, as for Malassez cells. In this case, holographic measurement and classification algorithms make it possible to measure between 10^2^ and 10^3^ cells. The measurement is considered to be more accurate because it is not dependent on the operator. Spectroscopy cuvettes are filled with 3 mL of cell suspension. The cuvettes are 10 mm long and the diameter of the optical beam is 3 mm, resulting in a measurement volume of 70 µL, corresponding to 7 × 10^4^ counted cells.

Even if special care is taken to homogenize the flask suspension, a small sample volume remains less representative of the actual contents of the culture flask than a large volume (approximately 200 times more for spectral measurements). The number of cells enumerated also plays a role in the accuracy and representativeness of the measurement. Spectral measurements allow for the enumeration of 70 times more cells than in the best case of automated counters. The apparent disadvantage is the large sample volume required for spectral measurements. However, the ultimate goal of this work is to integrate the method into a closed system environment for in situ, sampling-less, real-time measurements where sample volume is irrelevant. In fact, an accuracy of ±5% is quite acceptable in usual cell culture monitoring, especially if the method used has the potential to be performed without sampling with results given in real time.

Figure 4 surprisingly shows that even with a low R^2^ value, the concentrations are accurately measured. In fact, Equation (2) efficiently describes the shape of the absorption spectra when the concentration is high enough (above 1 × 10^5^ cell.mL^−1^). For low concentrations (below this value), the spectra flatten and Equation (2) is no longer able to fit them accurately, resulting in a low R^2^. However, the concentration can efficiently be determined at low concentrations even when the R^2^ is low (Figure 4). This shows that sub-function a1(C) is predominant in determining the concentration as it reflects the absorption amplitude rather than the whole shape. In fact, a method derived from Beer–Lambert could have been used, as we published previously [33], but it was preferred to determine the absorption spectra formula (Equation (2)) as it also allows for the determination of concentrations of mixtures [32] and other culture characteristics such as cell viability (not yet published).

### 4.4. Precaution to Be Taken When Fitting Expansion Folds

The folds extend over several orders of magnitude (1 to 10^4^) with data points regularly distributed over time. Fitting finds coefficients of a given equation that minimize the difference between the function and the data. For data spanning several orders of magnitude, the minimization will mainly result from the difference between the function and the very high value data points at the expense of the low-value data.

One way to overcome this inconvenience is to consider logarithmic values of the data. In this way, the fitting only concerns data that evolve over 1 order of magnitude, and no data point is favored. Therefore, fittings in Figure 7 were performed considering $“{log}_{N}(fold)”$ and the fitting function $“{log}_{N}\left(1+at^{b}\right)”$. Note that the log function can be considered in any base “*N*”. Table 8 shows the R^2^ obtained when fitting data corresponding to the concatenation of the three measurement methods either directly or using the ${log}_{N}$ transform (to be compared to Table 6).

### 4.5. Considerations About Instantaneous Generation Times

Our results show that primary T-cell populations do not expand exponentially, but do so according to a “power” dynamic. This implies that the concept of a unique/constant generation time is not valid as the proliferation rate slowly decreases with time. To account for this particular dynamic, Instantaneous Generation Times were proposed. These represent, at time “t”, the generation time thaT-cells potentially have at that particular moment. The term IGT could also be replaced by potential or even virtual generation time. This aspect is open to discussion and is beyond the scope of this paper.

IGTs decrease from D0 to approximately D1 (Figure 9). In fact, the IGT values at D0 and the minimum values of IGTs did not occur at the same time, which means that the T-cells were not at the same developmental stage when the measurements started at D0 (Table 9). In fact, they are donor-derived and therefore could differ slightly from one donation to another (which explains the different expansion folds and IGTs), which also applies to the T-cell preparation period before D0.

The slopes in Table 7 are not directly comparable to the final fold values in Table 5 since the latter also depend on the initial T-cell concentrations. The quantity Q (Equation (8)) can be defined to relate initial concentrations, slopes, and final fold values.

(8) Q=C(2)/slope

In Equation (8), *C(2)* represents the concentration at D2 when the suspensions contain mainly T-cells. A high Q value is associated with a high final fold (Table 10). This is always confirmed, except for donation #3. The latter showed an unexplained rapid increase in the fold value at D11, as shown in Table 10 (last row), where the percentage difference between the experimental fold value and the value fitted by Equation (4) is given.

In fact, a parabolic correlation between the Q value and the final fold indicates that the Q value represents an early marker of the final fold of the culture.

### 4.6. Power-Law Dynamic of Primary T-Cell Culture

The growth dynamics of primary T-cells have already been reported [35,36,37,38]. The expansion folds and concentration evolution of T-cells from healthy donors were presented in [36,37,38] and folds from patients in [35]. Both the folds and concentrations were plotted in a linear vertical scale, which makes it difficult to determine whether the growth in exponential or obeys to a power-law. Expansion folds plotted on a logarithmic vertical axis were reported in [39,40]. A very similar fold evolution can be seen [39], while the fold evolution is impressively linear over the 4-week experiments [40]. However, a mathematical description of the T-cell growth evolution does not seem to have been reported.

More generally, mathematical descriptions of cell expansion have been proposed. The Monod equation can be used to describe cell growth as a function of nutrient availability [41]. The logistic growth model (or sigmoid growth model) has been established to describe the entire growth process, including the initial exponential phase, the deceleration phase (when the cell population still increases but at a reduced rate), and the stationary phase (when the cell population remains constant) [42]. Another sigmoidal representation is used in the Gompertz model, which emphasizes the declining growth rate using a double exponential [43]. However, all of these models were developed to account for resource limitations (nutrients, space, etc.). This is not the case in the present study as cells were regularly resuspended in fresh volumes of culture medium.

Indeed, primary T-cells do not multiply indefinitely but are regulated to slow down and stop the multiplication. This implies that the growth rate decreases with time, which results in an increase in the generation time during the cell culture. The growth dynamic cannot be exponential because an exponential growth would imply a constant generation time. A power-law function describes better cell growth than an exponential one and this is independent of the absence of error bars in Figure 5 and Figure 6 (as the R^2^ indicated in Figure 7 shows). Indeed, fitting produces a smoothed representation of experimental data. If data are provided with error bars, the fitting will remain the same and only the value of the R^2^ coefficient will vary due to the value of the ‘residual sum of squares’ (SS_res_), according to data uncertainties. The more uncertain the data, the lower the R^2^. If a certain function “f” (the power-law function here) produces a better R^2^ than another function “g” (the exponential function here), the R^2^ attached to function “f” will always remain higher than the R^2^ attached to function “g”, regardless of the data uncertainties.

The power-law dynamics of the cell culture clearly indicate a mechanism for slowing the growth rate. The latter can follow a linear or power-law description [44]. Figure 9 shows that the linear deceleration growth rate mechanism is most likely. The deceleration phase is often defined as the phase between the exponential and stationary phases. However, Figure 6 and Figure 8 show that there is virtually no exponential phase. The mathematical description of the behavior of expansion folds could potentially be used to predict the final state of the T-cell culture. Note that the growth dynamics observed with CEM-C1 during 4 days of culture experiments were exponential [34].

### 4.7. Integration Possibility in a Closed-Loop, Real Time and Sampling-Less Device

The production of CAR T-cells, particularly the expansion phase, takes several days, with each additional day adding to the overall cost. Current quality control measures track cell expansion through frequent sampling, which increases the risk of contamination. Currently, several CAR T therapies are commercially available [45,46], but they come at a significant price due to the complexity of their production and administration. Accurately predicting the range of diseases these therapies could address or the number of patients who could benefit remains a significant challenge.

Automation of the CAR T-cell manufacturing process has the potential to significantly reduce costs by reducing the need for highly specialized labor and minimizing errors, thereby addressing the high costs associated with these treatments. In addition, automation could speed up the production cycle, allowing patients to receive therapy sooner and reducing the need for costly interim medical treatments. This approach could also reduce the burden on healthcare systems and increase the accessibility of CAR-T therapies.

For a few years, white light spectroscopy has been considered well suited for integration into online systems [4]. This closely aligns with the benefits of automating CAR T production. In addition, advances in compact light sources [47] and spectrometers as small as a fingernail [48] make the integration of this technology more feasible and efficient. An online, real-time monitoring system capable of tracking cell expansion, assessing cell quality, and detecting contamination in real time would be of significant value in advancing CAR T manufacturing systems.

The experiments shown here were performed using spectroscopy cuvettes. As for our previous studies, we presented proofs of concept [30,31,32]. The sampling-less integration can be made in different ways, as shortly mentioned in [3,4]. These methods can be easily included in production units like the Prodigy system from Miltenyi Biotec [49]. Integration may rely on the use of a derivation of the bioreactor content or a transmission dip probe [50]. A sampling-less, automated and real-time prototype is being fabricated and should be presented shortly. Also, the experiments were performed using successive dilutions to access expansion folds. This is a theoretical assumption and T-cell behavior may slightly differ in real conditions where culture volumes are of the order of a few hundreds of mL.

## 5. Conclusions

We presented culture experiments performed with T-cells from healthy donors. White light spectroscopy was used to measure T-cell concentrations. The equation describing spectra shapes as a function of concentration, which were established and used to measure suspension concentrations during 11 days of culture with accuracies around ±5%, comparable to those of more classical methods. The expansion folds showed thaT-cell growth was not exponential but followed a power-law dynamic. To our knowledge, this is the first mathematical demonstration of non-exponential dynamics of cultures. Since the generation time is not constant, the concept of Instantaneous Generation Times was proposed for the first time. Considerations on a possible predictive marker of the final culture concentration were proposed in the Discussion section.

In fact, white light spectroscopy has proven to be an ideal candidate for monitoring CAR T-cell production as it allows concentration and culture dynamics to be measured in a closed-loop system without sampling and monitoring results to be displayed in real time. This would greatly simplify the production process, drastically reduce the risk of contamination by eliminating the need for sampling, and directly reduce the cost of therapy, thereby increasing the number of eligible patients.

## Figures and Tables

**Table 1:** List of reagents and equipment.

Reagent	Supplier (Country)	Reference
T-cell TransAct^TM^ Human	Miltenyi (Bergisch Gladbach, Germany, supplier France)	130-111-160
RPMI	Corning (Glendale, AZ, USA, supplier France)	98-1507-LT
Human Serum (HS)	Internal product from French Blood Agency (France)	Batch: #2 du 2022-12-16
Penicillin/Streptomycin (PS)	EUROBIO (Les Ulis, France)	CABPES01-0U
IL2 (interleukin 2)	Clinigen (Yardley, PA, USA, supplier France)	03400956215867
Brilliant Violet 510^TM^ Anti-human CD45	SONY Biotechnology (San Jose, CA, supplier France)	2120180
PE Mouse anti human CD4	Becton Dickinson (Franklin Lakes, NJ, USA, supplier France)	555347
APC Mouse anti human CD8	Becton Dickinson (Franklin Lakes, NJ, USA, supplier France)	555369
7-AAD Viability Staining Solution	SONY Biotechnology (San Jose, CA, USA, supplier France)	2702020
**Equipment**	**Supplier (country)**	**Reference**
6-well plate	Corning (Glendale, AZ, USA, supplier France)	351146
T75 flask	Corning (Glendale, AZ, USA, supplier France)	353136
Malassez cell	NanoEntek (Seoul, Republic of Korea, supplier France)	DHC-M01 2M18222
Automated Cell Counter	Anvajo (Dresde, Germany, supplier France)	FluidLab R-300
Heraeus Cryofuge Centrifuge	Thermo Scientific (Waltham, MA, USA, supplier France)	5500i
Centrifuge	Eppendorf (Wesseling-Berzdorf, Germany, supplier France)	5702 R
Cytometer	Becton Dickinson (Franklin Lakes, NJ, USA, supplier France)	FACS Fortessa Flow Cytometer
Cytometer	Miltenyi (Bergisch Gladbach, Germany, supplier France)	Analyzer 16
White light source	Avantes (Apeldoorn, The Netherlands, supplier France)	Avalight-DH-S-BAL
Cuvette holder	Avantes (Apeldoorn, The Netherlands, supplier France)	CUV_UV/VIS
Spectrometer	Ocean Optics (Orlando, FL, USA, supplier France)	USB 4000 UV-VIS-ES
OceanView software	Ocean Optics (USA, supplier France)	N° 2.0.15
Calculation Software	MathWorks (Natick, MA, USA, supplier France)	Matlab^TM^ R2020b

**Table 2:** Days on which concentrations were measured (X: measurement day).

	D0	D1	D2	D3	D4	D5	D6	D7	D8	D9	D10	D11
**#1**	X	X	X	X	X			X	X		X	X
**#2**	X	X	X	X	X			X	X		X	X
**#3**	X	X	X	X	X			X	X		X	X
**#4**	X	X	X	X	X			X	X	X		X
**#5**	X	X	X	X	X			X	X	X		X
**#6**	X	X	X		X			X	X	X		X

**Table 3:** List of final parameters used in Equation (2).

Final Parameters	p1a1	b1	p1c1	p2c1	a2	b2	c2
**Value**	5.59 × 10^−7^	535.93	39.49	0.21	3.48	274.01	1281.6

**Table 4:** Dilution factors and days on which dilutions were performed.

Day	D0	D1	D2	D3	D4	D7	D8	D9	D11
**#1**	1	1	1	6.8	1	7.6	12	1	1
**#2**	1	1	1	6.45	1	7.45	20.5	1	1
**#3**	1	1	1	5.6	1	9.5	14.5	1	1.325
**#4**	1	1	1	7.05	1	9.95	5.75	1	1
**#5**	1	1	1	6.05	1	8.45	4.83	1	1
**#6**	1	1	2.5	-	13.5	14	9.5	1	2.03

**Table 5:** Average final folds for all experiments.

Donation	#1	#2	#3	#4	#5	#6
**Final fold value**	1889	2301	4279	740	544	13,994

**Table 6:** Power function parameters (spectrometry data).

Donation	#1	#2	#3	#4	#5	#6
a (day^−1^)	0.91	0.2	0.35	0.56	1.05	1.62
b (no unit)	3.14	3.81	3.71	2.97	2.49	3.74
R^2^	0.99	0.99	0.99	0.99	0.98	0.997

**Table 7:** Slopes of IGTs.

Donation	#1	#2	#3	#4	#5	#6
Slope (h.day^−1^)	6.5	5.8	5.8	7.3	8.5	5.4

**Table 8:** R^2^ obtained with or without the logarithm transform.

Donation	#1	#2	#3	#4	#5	#6
Without	0.94	0.94	0.89	0.94	0.81	0.96
With	0.99	0.99	0.99	0.99	0.98	0.997

**Table 9:** IGT minima times.

Donation	#1	#2	#3	#4	#5	#6
Initial value (h)	24.7	36.6	31.8	29.2	23.5	21.1
Min. time (h)	26.7	42.7	37.3	32	21.3	24

**Table 10:** Relations between slopes, final fold values and initial concentrations.

Donation	#1	#2	#3	#4	#5	#6
C(2)	1.7	1.55	1.39	1.74	1.6	3.23
Slope	6.5	5.8	5.8	7.3	8.5	5.4
Q	0.2615	0.2672	0.2397	0.2384	0.1882	0.5981
Final fold value	1889	2301	4279	740	544	13,994
% diff. at D11	11	8	28	−5	−15	−10

## Data Availability

Research data are available on demand to the corresponding author.
